# Comparative morphology and development of extra-ocular muscles in the lamprey and gnathostomes reveal the ancestral state and developmental patterns of the vertebrate head

**DOI:** 10.1186/s40851-016-0046-3

**Published:** 2016-04-14

**Authors:** Daichi G. Suzuki, Yuma Fukumoto, Miho Yoshimura, Yuji Yamazaki, Jun Kosaka, Shigeru Kuratani, Hiroshi Wada

**Affiliations:** Graduate School of Life and Environmental Sciences, University of Tsukuba, 1-1-1 Tennodai, Tsukuba, Ibaraki 305-8572 Japan; Laboratory for Evolutionary Morphology, RIKEN, Kobe, 650-0047 Japan; Department of Cytology and Histology, Okayama University Graduate School of Medicine, Dentistry and Pharmaceutical Sciences, 2-5-1 Shikata-cho, Okayama, 700-8558 Japan; Sumitomo Besshi Hospital, 3-1 Oji-cho, Niihama, Ehime 792-8543 Japan; Graduate School of Science and Engineering for Research, University of Toyama, 3190 Gofuku, Toyama, 930-8555 Japan; Center for Medical Science, International University of Health and Welfare, 2600-1 Kitakanemaru, Ohtawara, Tochigi 324-8501 Japan

**Keywords:** Evo-devo, Extra-ocular muscles, Lamprey, Head mesoderm, Head segmentation

## Abstract

The ancestral configuration of the vertebrate head has long been an intriguing topic in comparative morphology and evolutionary biology. One peculiar component of the vertebrate head is the presence of extra-ocular muscles (EOMs), the developmental mechanism and evolution of which remain to be determined. The head mesoderm of elasmobranchs undergoes local epithelialization into three head cavities, precursors of the EOMs. In contrast, in avians, these muscles appear to develop mainly from the mesenchymal head mesoderm. Importantly, in the basal vertebrate lamprey, the head mesoderm does not show overt head cavities or signs of segmental boundaries, and the development of the EOMs is not well described. Furthermore, the disposition of the lamprey EOMs differs from those the rest of vertebrates, in which the morphological pattern of EOMs is strongly conserved. To better understand the evolution and developmental origins of the vertebrate EOMs, we explored the development of the head mesoderm and EOMs of the lamprey in detail. We found that the disposition of lamprey EOM primordia differed from that in gnathostomes, even during the earliest period of development. We also found that three components of the paraxial head mesoderm could be distinguished genetically (premandibular mesoderm: *Gsc*+/*TbxA*–; mandibular mesoderm: *Gsc*–/*TbxA*–; hyoid mesoderm: *Gsc*–/*TbxA*+), indicating that the genetic mechanisms of EOMs are conserved in all vertebrates. We conclude that the tripartite developmental origin of the EOMs is likely to have been possessed by the latest common ancestor of the vertebrates. This ancestor’s EOM developmental pattern was also suggested to have resembled more that of the lamprey, and the gnathostome EOMs’ disposition is likely to have been established by a secondary modification that took place in the common ancestor of crown gnathostomes.

**Electronic supplementary material:**

The online version of this article (doi:10.1186/s40851-016-0046-3) contains supplementary material, which is available to authorized users.

## Introduction

The morphological nature and ancestral configuration of the vertebrate head are longstanding topics of interest in comparative morphology and evolutionary biology. A peculiar component of the vertebrate head is the extra-ocular muscles (EOMs), which control the visual field by the moving eyes. These muscles are derived from the paraxial portion of the head mesoderm, and their development has attracted the attention of morphologists in the context of head mesodermal segmentation.

**Fig. 1:**
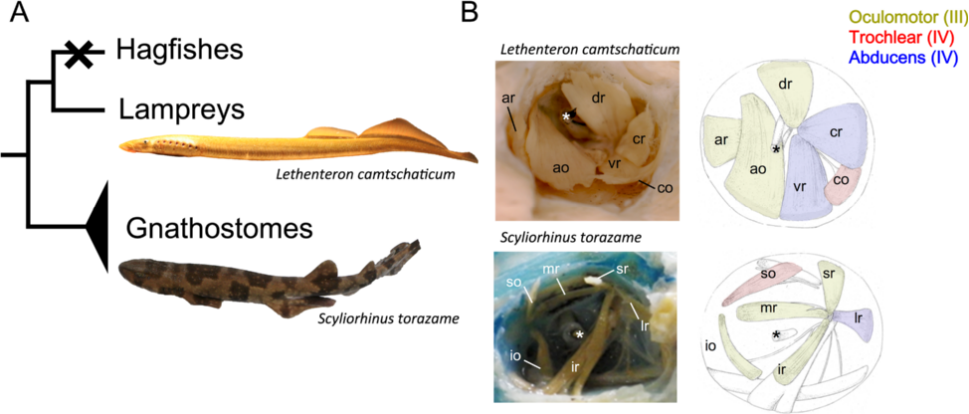
Phylogenetic relationships among vertebrates and gross anatomy of extra-ocular muscles. **a** A phylogenetic tree of the vertebrates. The hagfishes lack extra-ocular muscles. **b** Gross anatomy of the extra-ocular muscles of the lamprey *(Lethenteron camtschaticum*) and shark (*Scyliorhinus torazame*). Asterisks indicate the optic nerve. The extra-ocular muscles are colored based on their innervation nerve: the oculomotor (III), yellow, trochlear (IV), red; abducens (IV), blue, respectively

Some researchers believe that these muscles are derived from rostral mesodermal segments of an amphioxus-like ancestor (reviewed in [1–4]). This idea partially stems from the presence of the epithelialized head mesodermal cysts (head cavities) in the shark embryo [5]; the head mesoderm in elasmobranch embryos typically forms three pairs of head cavities, from anterior to posterior, the premandibular (pc), mandibular (mc), and hyoid cavities (hc). These cavities later differentiate into six EOMs innervated by three cranial nerves: the oculomotor (III), trochlear (IV), and abducens nerves (VI) (Fig. 1). Although a complete set of head cavities arises only in cartilaginous fishes, they are occasionally found in osteichthyans including amniotes [6–15]. This indicates that possession of these cavities is a shared, derived characteristic for gnathostomes, although they tend to disappear in many osteichthyan taxa.

In avians, the head cavities have disappeared except for the remnant premandibular cavity [16–18]. In a different line of studies, a series of pseudosegmental blocks, so-called “somitomeres”, have been detected by scanning electron microscopy in the early head mesoderm, in which no head cavities are expected to arise (reviewed in [17]). Although the existence of the somitomeres has been questioned (reviewed in [3]), the EOMs do develop from a part of the head mesoderm corresponding to the sites of head cavities in elasmobranchs [11, 18–24].

Classically, the head mesoderm of lampreys, which belong to the most basal group of vertebrates (cyclostomes), was also thought to be segmented along the anteroposterior axis, similar to that in the head cavities in elasmobranch embryos [25–27], and EOMs were thought to differentiate from the three head cavities, innervated by their respective motor nerves [25, 26]. However, scanning electron microscopy-based observations of the Arctic lamprey, *Lethenteron camtschaticum* by Kuratani et al. [28], show no signs of segmental boundaries in the dorsal (paraxial) head mesoderm. Rather, the dorsal head mesoderm was simply secondarily regionalized into preotic and postotic portions by the otic vesicle, and the ventral part segmented passively by the pharyngeal pouches, in accordance with the notion of branchiomery proposed by Sewertzoff [29]). This finding suggests that the ancestral head mesoderm of the vertebrates is likely to have been unsegmented in the paraxial portion, raising the question of how the EOMs developed from this unsegmented head mesoderm.

The anatomical disposition and the innervation patterns of EOMs are highly conserved among gnathostomes, so much so that Neal [26] once noted that “[t]heir ‘evolutionary potential’ appears to be approximately zero”. However, the patterns of lamprey EOMs have been known to differ from living gnathostomes (Fig. 1, [30–32]). In the latter, the oculomotor nerve innervates four of the EOMs (medial rectus (mr), superior rectus (sr), inferior rectus (ir), and inferior oblique (io)), whereas the trochlear and abducens innervate only single EOMs (superior oblique (so) and lateral rectus (lr), respectively). In contrast, the lamprey oculomotor nerve innervates only three EOMs (anterior rectus (ar), dorsal rectus (dr), and anterior oblique (ao)), while the abducens innervates two (ventral rectus (vr) and caudal rectus (cr)). Furthermore, the caudal oblique (co) muscles of lampreys, which are innervated by the trochlear nerve, attach to the orbit far more caudally than do those of modern gnathostomes. Because the EOMs have degenerated completely in hagfishes [32], the lamprey is the only key extant animal to speculate ancestral state of the EOMs and its developmental mechanisms.

To understand the evolutionary origin of the vertebrate EOMs and suggest a possible ancestral state of the vertebrate head, we examined the development of the embryonic head mesoderm and EOMs in lampreys. We found that the developmental pattern of EOMs was also conserved in the lamprey, because the muscle originated from three domains along the anteroposterior axis in the dorsal (paraxial) head mesoderm. However, EOM disposition was different between lampreys and gnathostomes, as soon as EOMs were observed as differentiated muscles. These findings indicate that the developmental mechanisms of EOMs from the three subdivisions of the head mesoderm was already established in the common ancestor of vertebrates, and that diversification of the muscle patterns is due to changes during the later phase of development. Based on these findings, we discuss the ancestral state of the vertebrate dorsal head mesoderm and its differentiation.

## Materials and methods

### Animals

This study was performed in accordance with the Regulations on Animal Experimentation at University of Tsukuba. Approval is not needed for experimentation on fishes under Japanese law, Act on Welfare and Management of Animals.

Adult lampreys (*Lethenteron camtschaticum*, synonym *L. japonicum*) were collected from the Shiribeshi-Toshibetsu River, Hokkaido, Japan. The animals were anesthetized in ethyl,3-aminobenzoate methanesulfonate (MS-222). Mature eggs were squeezed from females and fertilized in vitro by sperm. Embryos were cultured at 16 °C, fixed in 4 % paraformaldehyde in 0.1 M phosphate-buffered saline (PBS) overnight, dehydrated in a graded methanol series, and stored in 100 % methanol at −20 °C. Developmental stages were determined as described by Tahara [33].

As ammocoete larvae were not readily available for *L. camtschaticum*, we used larvae from *Lethenteron* sp*.* N, related species of *L. camtschaticum* [34, 35]. These larvae were collected from the Kamo River, which flows through the middle of the Shougawa River, Toyama, Japan, in September.

Fertilized eggs of the cloudy catshark (*Scyliorhinus torazame*) were obtained from adults that were bred at 16 °C in seawater tanks. Shark embryos were staged following Ballard’s staging of *Scyliorhinus canicula*, a species closely related to *S. torazame* [36, 37].

### Histological analyses

*Lethenteron* sp. N and *Scyliorhinus torazame* were fixed in Bouin’s or Serra’s fixative, dehydrated, and embedded in paraffin. Sections were cut at a thickness of 6 μm and stained with hematoxylin and eosin, following a standard protocol.

### 3D reconstruction

The stained sections of *Lethenteron* sp. N and *S. torazame* were digitized using an Olympus BX60 microscope equipped with an Olympus DP70 camera and the Olympus DP controller software (Olympus, Tokyo, Japan). On the digitized sections, each anatomical component was colored and reconstructed using the Avizo 3D Visualization Framework (Maxnet Co., Ltd, Tokyo, Japan).

### Whole-mount immunofluorescence

Whole-mount immunofluorescence with anti-acetylated tubulin (Sigma, T6793) and anti-tropomyosin (Hybridoma bank, CH1) antibodies was performed according to the protocol described by Kuratani et al. [38] with some minor modifications. Briefly, fixed embryos stored in methanol were washed in TBST containing 5 % dimethylsulfoxide (TSTd). The embryos were then blocked with 5 % non-fat dry milk in TSTd (TSTM). They were incubated with the primary antibody (diluted 1:1,000 in TSTM) for 2–4 days at room temperature (RT). After washing with TSTd, samples were incubated with a secondary antibody (Invitrogen, Alexa fluor 555, A21424) diluted 1:200 in TSTM. After a final wash in TSTd, embryos were dehydrated and clarified in a 1:2 mixture of benzyl alcohol and benzyl benzoate (BABB) and then examined using a confocal laser microscope (LSM 510, Zeiss). The data were colored and projected by using a computational graphics editor (Photoshop CS6).

### Whole-mount and section in situ hybridization

*Gsc* was amplified from *L. camtschaticum* by RT-PCR from stage 25 specimens using the primers designed for *Petromyzon* genes (*PmGsc*, HQ248103) [39]. For the other genes, probes were synthesized by using the plasmids in accordance with previously described protocols (*PitxA*: Uchida et al. [40]; *MrfA*, *MA2*: Kusakabe et al. [41]; *Tbx1/10*: Tiecke et al. [42]). Whole-mount in situ hybridization was performed according to the protocol of Ogasawara et al. [43] with minor modifications. For section in situ hybridization, larval lampreys (*L.* sp. N) were fixed for three days in 4 % paraformaldehyde in 0.1 M phosphate-buffered saline (PBS), dehydrated, and embedded in paraffin. Sections were cut at a thickness of 8 μm. After washing out the paraffin, in situ hybridization for cryosectioned materials was performed following the protocol for whole-mount in situ hybridization, except that Tween 20 was not used at any step and proteinase treatment was omitted before hybridization.

### Cell labeling

St. 21 embryos were injected with 1 mM DiI, DiD, and DiO solutions (Vybrant Multicolor Cell-labeling kit, Molecular Probes). The embryos were excised from the egg membranes and placed in wells made in solidified agar on a plastic dish. Injections were performed with a fine glass pipette. The embryos were incubated for 10 days until st. 27 was approximately reached and were fixed in 4 % paraformaldehyde in PBS. Observation was made with a fluorescence microscope or confocal microscope (LSM510, Zeiss, Goettingen, Germany).

## Results

### Development of the EOMs and their innervation

**Fig. 2:**
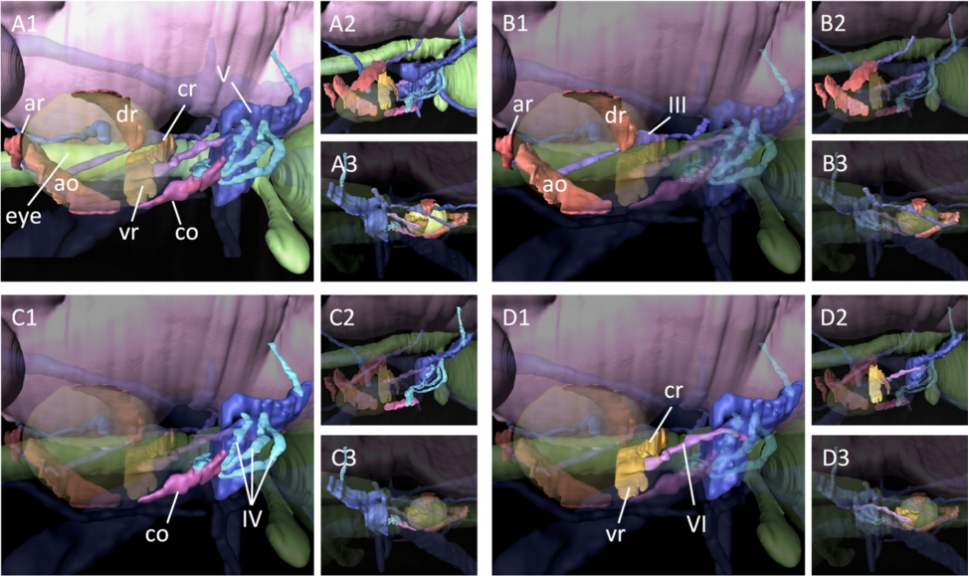
3D reconstruction of a lamprey ammocoetes larva. **a** Overview. **b** The oculomotor nerve and its innervating extra-ocular muscles. **c** The trochlear nerve and its innervating caudal oblique muscle. **d** The abducens nerve and its innervating extra-ocular muscles. **a1**–**d1**: Lateral; **a2**–**d2**: Dorsal; **a3**–**d3**: Medial view

To clarify the disposition and innervation pattern of EOMs in lamprey larvae, we first performed 3D reconstruction of a ca. 100 mm ammocoete larva (Fig. 2a–d). At this stage, six EOMs were already differentiated as distinct muscle primordia attached to the surface of the eye (Fig. 2a). They consisted of four rectus muscles (ar, dr, cr, and vr) and two oblique muscles (ao and co). This muscle organization was the same as that known in the adult lamprey (Fig. 1b, [30–32]). To confirm the muscle identities, we also analyzed the innervation patterns of these EOMs. As reported for adult specimens (Fig. 1b, [30–32]), the oculomotor nerve (III) innervated the ‘ar’, ‘dr’, and ‘ao’ muscles (Fig. 2b), the trochlear nerve (IV) the ‘co’ muscle (Fig. 2c), and the abducens nerve (VI) the ‘vr’ and ‘cr’ muscles (Fig. 2d). Notably, the pathways of the trochlear and abducens nerves partially overlapped those of the trigeminal nerve (V), and the trochlear nerve ramifies into sub-bundles and become fasciculated again near its terminus (Fig. 2c). We confirmed this observation by immunofluorescence analysis using an anti-acetylated tubulin antibody in early larvae, as described below. Furthermore, the attachment site of the ‘ao’ muscle to the cartilaginous orbital wall was relatively more ventral (Fig. 2b) than that in the adult, in which the ‘ao’ muscle crossed over the ‘ar’ muscle (Fig. 1).

**Fig. 3:**
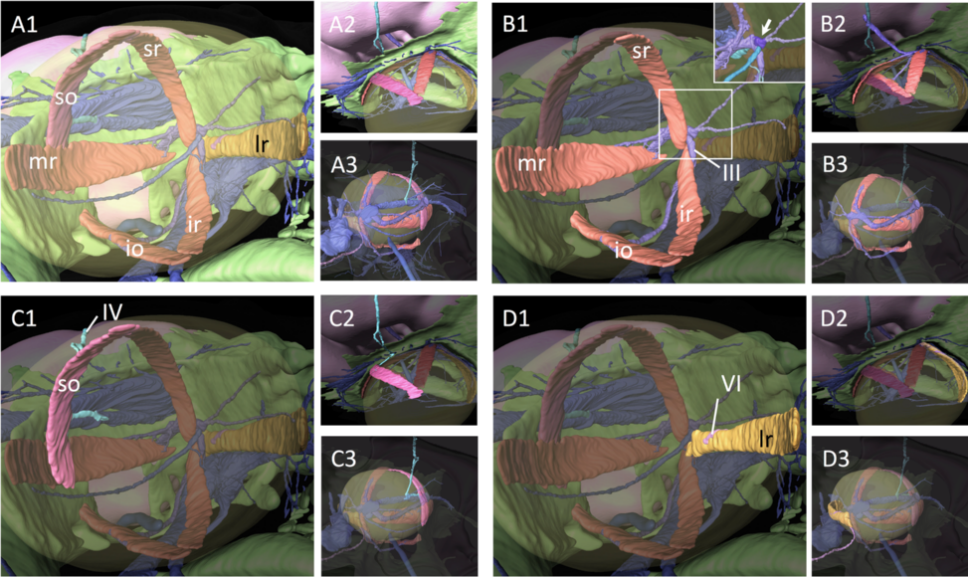
3D reconstruction on *Scyliorhinus torazame*. **a** Overview. **b** The oculomotor nerve and its innervating extra-ocular muscles. **a** detailed structure of oculomotor nerve (III) is shown in the inset, in which a ciliary ganglion-like structure is indicated by an arrow and highlighted in blue color. **c** The trochlear nerve and its innervating superior oblique muscle. **d** The abducens nerve and its innervating lateral rectus muscle. **a1**–**d1**: Lateral; **a3**–**d2**: Dorsal; **a3**–**d3**: Medial view

For the comparison, we constructed 3D images of a pre-hatching stage (72 mm long) embryonic shark (*Scyliorhinus torazame*) to represent gnathostomes (Fig. 3a–d). Consistent with the adult anatomy (Fig. 1b) as well as previous descriptions [30–32], the oculomotor nerve (III) innervated the ‘mr’, ‘sr’, ‘ir’, and ‘io’ muscles (Fig. 3b), the trochlear nerve (IV) the ‘so’ muscles (Fig. 3c), and the abducens nerve (VI) the ‘lr’ muscles (Fig. 3d). The ciliary ganglion was observed in the orbit (inset in Fig. 3b1), but no similar ganglion was found in the lamprey (Fig. 2).

**Fig. 4:**
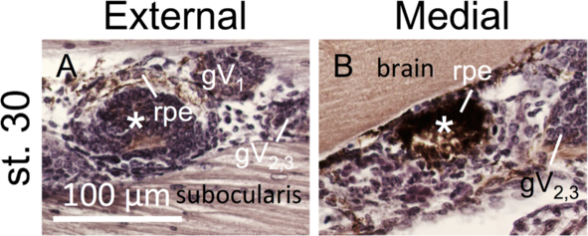
Histological analysis by the hematoxylin and eosin (HE) staining on the extraocular muscles in st. 30 prolarva. Asterisks indicates the eye. **a** External section. **b** Medial section

**Fig. 5:**
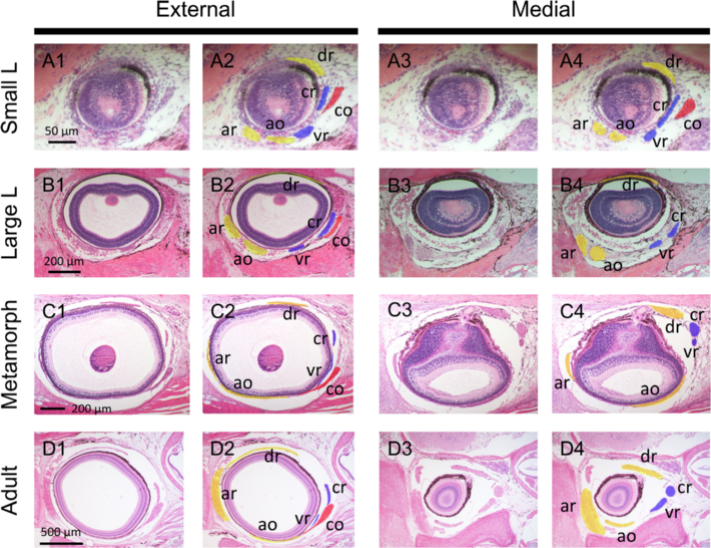
Histological analysis by hematoxylin-eosin (HE) staining of the extraocular muscles in larval, metamorphic and adult lampreys. **a** Small larva (3.5 cm, about half a year old). **b** Large larva (10 cm). **c** Metamorphic lamprey. **d** Adult lamprey **a1**–**d1**: External sections; **a2**–**d2**: External sections, colored; **a3**–**d3**: Medial sections; **a4**–**d4**: Medial sections, colored

To determine whether the disposition of the lamprey EOMs changes during development, we performed a histological analysis by hematoxylin and eosin (HE) staining in stage (st.) 30 prolarvae (Fig. 4), 35 mm (about half a year old, Fig. 5a),100 mm larvae (Fig. 5b), metamorphic (Fig. 5c) and adult lampreys (Fig. 5d). In st.30 prolarvae, we found no muscle fibers suggestive of EOM differentiation, but only mesenchymal cell masses surrounding the eyeball (Fig. 4).

In the 35 mm larvae, EOMs were found as fibrous, distinguishable six-cell clusters (Fig. 5a), identities of which were obvious from their disposition. Rectus muscles were located at the anterior, dorsal, ventral, and caudal parts in the orbit; thus, we named them the anterior, dorsal, ventral, and caudal rectus muscles, respectively. As for two oblique muscles, one primordium originated slightly ventral to ‘ar’, and was directed caudally; it was therefore identified as the ‘ao’ muscle. The other, identified as the ‘co’, originated from the dorsocaudal region in the orbit, was directed ventrally.

In the 100 mm larvae, the EOMs become more clearly compartmentalized and discriminable (Fig. 5b), suggesting the growth of the EOMs of this animal during the larval period, ranging 4–5 years. The topological disposition of the EOMs was the same as that in the 35 mm larvae. In the metamorphic stage, the external part of the EOMs became thinner and wider (Fig. 5c1), suggesting rigid attachment to the eyeball to exert its functional movement. The relatively immature state of larval EOMs may be due to the larval life style of this animal, in which the eyes do not possess image-forming vision [44–47]. Through all of the stages examined, the positions of the EOMs did not show radical changes, and it seemed likely that the EOM morphological pattern is established during the pre-metamorphic stages. However, the little change in the relationship between the ‘ar’ and ‘ao’ muscles was notable. During the larval period, these muscles at first run in parallel to each other (Fig 2a4, Bb), and cross each other in the adults (Figs. 1b and 5d4). This change is likely to occur during metamorphosis.

### Developmental mechanism of EOMs and patterning of head mesoderm

**Fig. 6:**
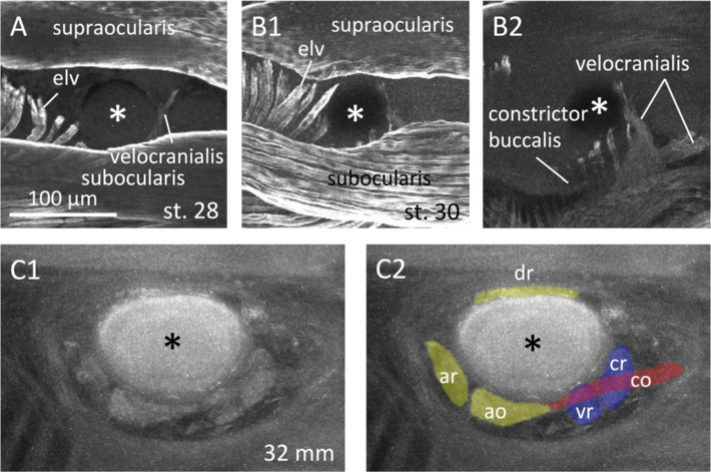
Whole-mount immunofluorescence with an anti-tropomyosin antibody. Asterisks indicates the eyes. **a** st. 28 prolarvae. **b** st. 30 prolarvae (**b1**: Overview; **b2**: Medial). **c** 32 mm larva (**c1**: Raw; **c2**: Colored)

To trace further the developmental origin of the lamprey EOMs, immunofluorescence analysis was performed using an anti-tropomyosin antibody in younger lamprey larvae (Fig. 6a–c). We did not detect any EOMs in st. 28 or st. 30 prolarvae, but did detect other muscles, including somatic/branchial muscles; supraocularis, subocularis, elevator labialis ventralis (elv), velocranialis, and constrictor buccalis (Fig. 6a, b, see also [48]).

**Fig. 7:**
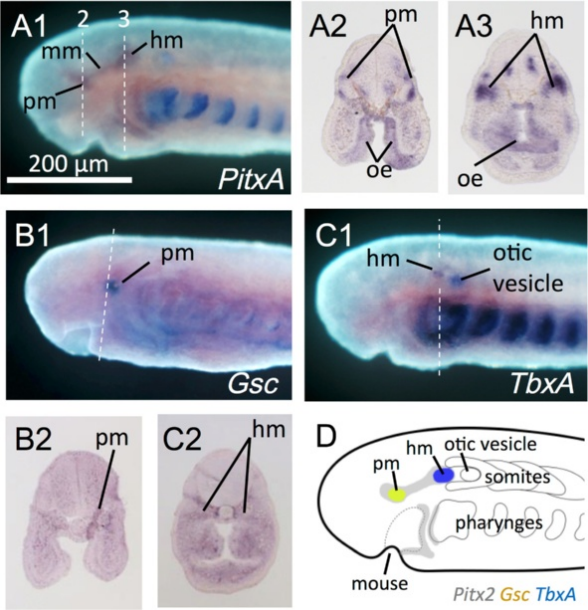
Whole-mount in situ hybridization in st. 26 lamprey prolarvae. A *PitxA* (**a1**: Lateral view; **a2**, 3: Sections). **b** *Gsc* (**b1**: Lateral view; **b2**: Section). **c** *TbxA* (**c1**: Lateral view; **c2**: Section). **d** Schematic illustration of the *PitxA*, *Gsc*, and *TbxA* expression patterns

**Fig. 8:**
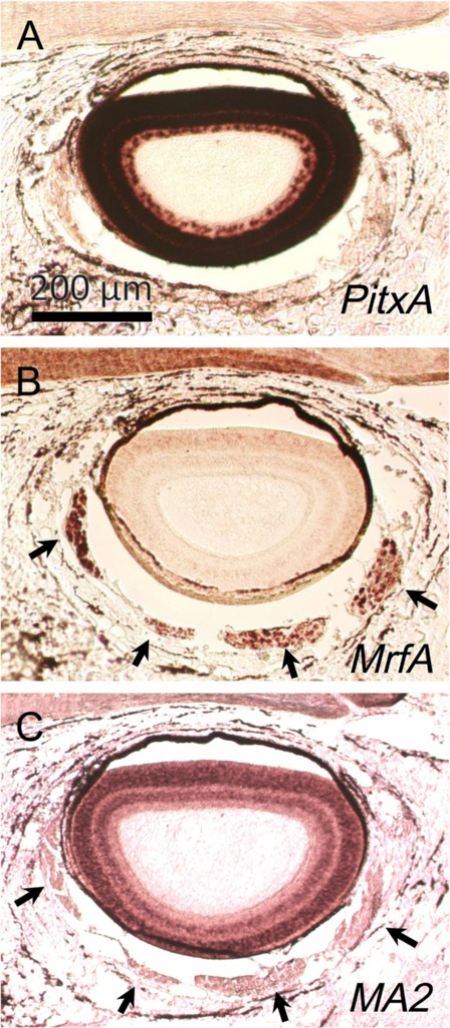
Sections in situ hybridization in 9 cm ammocoete larvae. **a** *PitxA*.**b** *MrfA*. **c** *MA2*

Next, we traced the developmental origin of EOMs by analyzing more upstream regulatory genes for EOMs. In gnathostomes, the genetic cascade involved in the development of EOMs has already been reported; genes encoding muscle-related factors (MRFs) act as determination and differentiation genes, *Pitx2* acts upstream of MRFs in cranial muscle progenitor cells, and *Pitx2-*null embryos lack EOMs [49]. This cascade is also conserved in sharks, in which *Pitx2* and *Myf5* (a member of the MRF family) are expressed in developing head mesoderm/cavities [50]. In a st. 26 prolarva, although *MrfA* (a member of the MRF family) and *MA2* (a muscle differentiation marker) were not expressed [41], we detected *Pitx2* transcripts in the head mesoderm (Fig. 7a). In contrast, in the 90 mm ammocoete larvae, *MrfA* and *MA2* were expressed in EOM prmordia, while the *Pitx2* expression ceased (Fig. 8).

Furthermore, we found that there was distinct genetic regionalization in the dorsal head mesoderm. In gnathostomes, *Gsc* is expressed in the prechordal plate [51], from which the premandibular mesoderm (pm) is thought to arise (lampreys: [28]; sharks: [36]). *Gsc* plays a dominant role as an organizer in head formation, including head muscle differentiation [52]. We found that *Gsc* was expressed in the anteriormost head mesoderm in the st. 26 lamprey prolarvae (Fig. 7b), and expression corresponded to that in the premandibular mesoderm. Simultaneously, *TbxA* transcripts were detected in the paraxial head mesoderm located anterior to the otic vesicle (Fig. 7c). In sharks, an equivalent expression has been observed in the hyoid cavity [50]. In the mouse, *Tbx1* (homolog of the lamprey *TbxA*) regulates craniofacial myogenesis [53]. Thus, *TbxA* expression in the lamprey is expected to represent that in the paraxial portion of the hyoid mesoderm (hm). These results suggest that the dorsal head mesoderm of lamprey, characterized by *PitxA* expression along the anteroposterior axis, is further specified through expression of *Gsc* and *TbxA*, i.e., pm: *Gsc*+, *TbxA*-; mm: *Gsc*-, *TbxA*-; hm: *Gsc*-, *TbxA*+ (Fig. 7d).

### Developmental lineage of the head mesoderm: origin of the differentiated EOMs

**Fig. 9:**
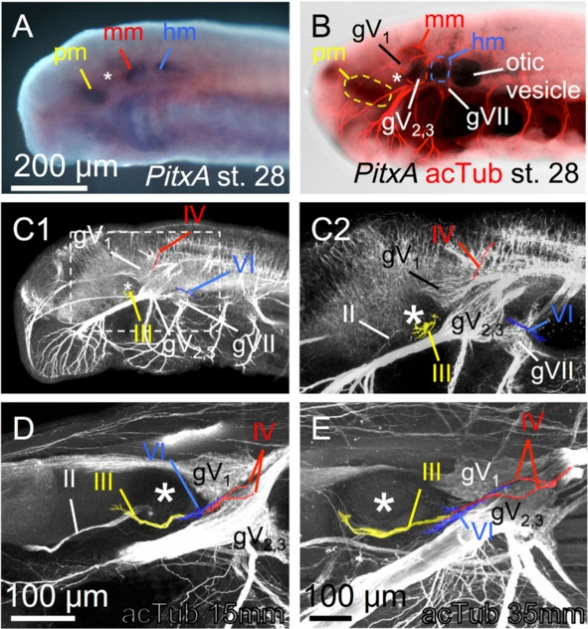
Nerves innervating head mesodermal portions and EOMs. Asterisks indicate the eyes. **a** *PitxA* expression in st. 28. **b** Double-staining of *PitxA* in situ hybridization (bright field) and an anti-acetylated tubulin antibody immunofluorescence (red). **c**, **d** Single immunofluorescence with the anti-acetylated tubulin antibody. **c** st. 28 (**c1**: Overview; **c2**: magnified dashed box region in **c1**). **d** 15 mm larva. **e** 35 mm larva

On examination of expression of *Pitx*, *Gsc*, and *Tbx*, three distinct domains were identifiable in the lamprey dorsal head mesoderm, in a pattern similar to those in gnathostomes. Thus, via immunofluorescence analysis using an anti-acetylated tubulin antibody, we examined differentiation of the three lamprey head mesodermal portions into the specific EOM groups innervated by the respective cranial motor nerves as seen in shark head cavities [5]. In the st. 28 prolarva, although the head mesoderm was not differentiated into the EOMs (Fig. 6a), *PitxA* was expressed in the three components of the dorsal head mesoderm (Fig. 9a). At this stage, the EOM-innervating nerves were already extending their fibers, and the distribution pattern corresponded to each portion of the dorsal head mesoderm: the oculomotor nerve (III) reached the premandibular mesoderm, the trochlear nerve (IV) the mandibular mesoderm, and the abducens nerve (VI) the hyoid mesoderm (Fig. 9b, c). These fibers approached the orbit in the 15 mm larvae (Fig. 9d), and their distribution pattern was maintained through the larval period, by which time the EOMs had already been formed (35 mm; Fig. 9e, see also Fig. 6c). These results indicate that the three components of the dorsal head mesoderm are assigned morphologically to respective nerves in a modern gnathostome pattern, and that nerve innervation is maintained through differentiation into EOMs, supporting differentiation of the specific paraxial head mesodermal portion into specific subsets of EOMs.

**Fig. 10:**
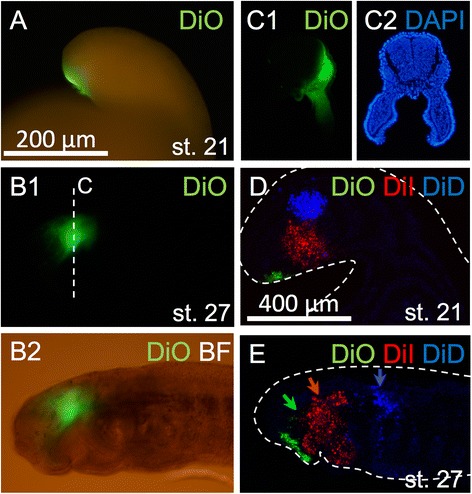
Dye injections on the head mesoderm. **a** DiO injection into the premandibular mesoderm of the st. 21 embryo. **b** DiO injected sample in st. 27 (**b1**: DiO fluorescence; **b2**: DiO fluorescence and bright field). DiO fluorescence is observed in the periocular region. **c** Section in the dashed line plane in **b1** (**c1**: DiO fluorescence; **c2**: DAPI fluorescence). **d** Three-color dye injections on the three mesodermal portions (premandibular; DiO, mandibular; DiI, hyoid; DiD, respectively). **e** Dye injected sample in st. 27. The three mesodermal portions retained their cohesion

In lamprey development, the premandibular mesoderm is derived from the prechordal plate, and the mandibular and hyoid mesoderm are regionalized rostrocaudally from each other by the growth of the first pharyngeal pouch [28]. Based on our results (Fig. 7), each of these subdivisions appears to correspond to a genetically-specified subdivision, as described above. However, there is another possibility that the mesenchymal cells are mixed and then become re-specified. Thus, we performed cell-labeling experiments to determine whether each head mesodermal portion retained its cohesion from its origin or became mixed. First, only DiO was injected into the prechordal plate region in st. 21 embryos (Fig. 10a) and incubated until st. 27. At st. 27, a DiO signal was observed around the eyeball, although the eyeball itself was also labeled as an artifact (Fig. 10b, c). Subsequently, triple dye injections were performed; DiO was injected into the prechordal plate, DiI into the mandibular mesoderm, and DiD into the hyoid mesoderm in st. 21 embryos (Fig. 10d). The mesodermal portions retained their cohesion and did not mix with each other in almost all of the larvae at st. 27 (*n* = 43/48; no fluorescent signal was detected in the remaining 5 samples), (Fig. 10e). The positions of these mesodermal portions also corresponded to the expression patterns of *Gsc* and *TbxA* as described above (Fig. 7d, e). These results indicate that the above noted dorsal head mesoderm is regionally specified early in development as well, with respect to their developmental fates.

## Discussion

### Developmental mechanism of the EOMs and the head mesoderm

The aim of the present study was to determine the evolutionary history of vertebrate EOMs. First we compared the disposition and nerve innervation of the EOMs between larval lampreys and shark embryos. The results showed that the disposition and nerve innervation patterns were quite different between these two animals (Figs. 2 and 3). We then traced the developmental process of the EOMs in lampreys using histological analysis. The overall disposition of the lamprey EOMs is established as early at 32 mm larvae (approximately six-months-old, Fig. 6c) in the paraxial (dorsal) head mesoderm, which is regionally and genetically specified during early stages of development (Figs. 4, 5 and 6). These results indicate that the lampreys and gnathostomes show different distributions of EOMs as soon as they are observed as differentiated muscles (lamprey: Fig. 6c; chick: reviewed in [22]).

In contrast, we found that the genetic cascade involved in development seems to be conserved in the lamprey, and that the dorsal head mesoderm is first marked by the expression of *PitxA* (at st. 26, Fig. 7). Furthermore, the expression patterns of *Gsc* and *TbxA* (Fig. 7) suggest that regionalization as well as specification of the three mesodermal portions underlie the distinct genetic characterizations. *Gsc* expression in the premandibular region is also observed in the zebrafish [54] and mouse [55], and *Tbx* expression has been observed in the dorsal hyoid region of the shark [50], zebrafish [56], *Xenopus* [57], chick [22], and mouse [58]. Thus, these expression patterns appear to be conserved among vertebrates. In addition, these mesodermal portions retain their cohesion and attract their respective innervating nerves (Figs. 9 and 10), similar to those in the shark head cavities [5], even if there is no morphological segmentation in the lamprey dorsal head mesoderm [28].

Since we did not detect any muscle differentiation markers such as the *MA2* gene or the anti-tropomyosin antibody during the developmental stage at which *Pitx*, *Gsc* and *Tbx* expressions were detected, it was unclear whether the *Pitx*-positive head mesoderm truly differentiated into EOMs in the lampreys. We circumvented this problem by examining head mesodermal innervation by motor nerves. We present evidence indicating that, although the muscle markers were not detected at the time *Pitx* expression was detected, motor innervation was observed at this stage. The oculomotor nerve fibers reached the premandibular mesoderm, the trochlear nerve the mandibular mesoderm, and the abducens nerve the hyoid mesoderm (Fig. 9b, c). This innervation pattern supported the hypothesis that lamprey EOMs differentiate from the three components of the dorsal head mesoderm.

**Fig. 11:**
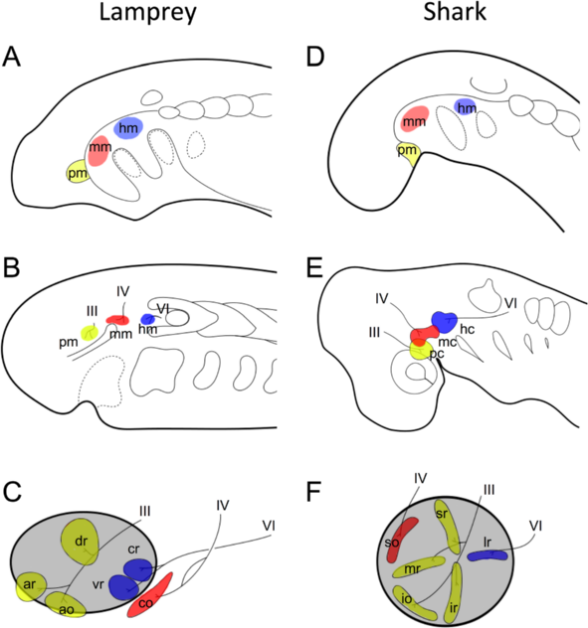
Schematic illustration of the comparison of the EOMs development. **a**–**c** lamprey. **d**–**f**: shark. **a**, **d** Pharyngeal stages. **b**, **e** Three head mesodermal portions innervated by respective motor nerves. **c**, **f** Differentiated state

**Fig. 12:**
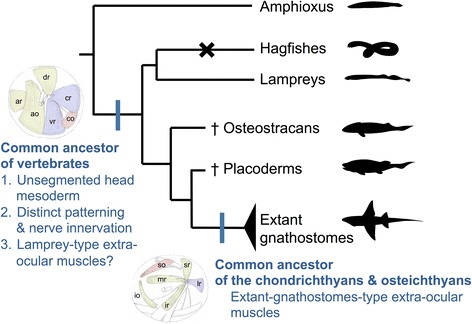
Hypothetical scenario for the evolution of the EOMs. In the common ancestor of the vertebrates had unsegmented head mesoderm but there were three mesodermal portions with distinct genetic patterning and motor nerve innervation. These had lamprey-type EOMs, which was conserved in Osteostracans and Placoderms (in hagfishes, EOMs are completely degenerated). In the common ancestor of the crown gnathostomes, the disposition of the EOMs changed to the extant-gnathostome-type

Figure 11 shows a comparison of EOM development in lamprey and shark. In both species, the early head mesoderm is primarily uniform with no overt segmental patterns at paraxial levels (Fig. 11a, d, [28, 36]). However, it is specified into three components, each innervated by a single cranial nerve (cranial nerves III, IV, and VI; Fig. 11b, e). In the shark, these components correspond to three pairs of epithelial coeloms called head cavities [36]. Finally, the EOMs are formed in their lineage-specific dispositions (Fig. 11c, f). This comparison indicates that the derivation of EOMs from the three components of dorsal head mesoderm would have already been established by the latest common ancestor of vertebrates (Fig. 12).

### Ancestral state of EOMs in vertebrates and subsequent evolutionary modifications in gnathostomes

The comparison of EOM development between lampreys and gnathostomes described above indicated that the evolutionary modification of EOMs would have been introduced into a developmental stage following establishment of the three dorsal head mesodermal portions, but preceding the start of muscle differentiation (corresponding to between st. 28 and the 35 mm early larval stage of lampreys). However, the type of EOM distribution that represents the ancestral disposition of the vertebrates could not be determined in the present study, as there are only two groups with different EOM patterns (lampreys and extant gnathostomes), and both hagfishes and the outgroup (protochordates) lack EOMs. Nevertheless, previous studies on fossil records [59–62] analyzed the morphology of the orbit including distribution of myodomes (orbital wall depressions indicating muscle insertions) and suggested that the disposition of EOMs in osteostracans and placoderms would have been similar, to some extent, to that of lampreys than the extant gnathostomes. Based on recent phylogenetic analyses [63–65], the pattern of EOM disposition common to chondrichthyans and osteichthyans would have been established as a synapomorphy of jawed vertebrates in some lineages of placoderms (Fig. 12). This modification may be functionally linked with the postorbital connection between the palatoquadrate and neurocranium, which is a synapomorphy of the crown gnathostomes [61]. As the caudal oblique muscle could interfere with the postorbital connection between the palatoquadrate and neurocranium, the position of the caudal oblique muscle may have become anterior within the orbit.

Based on the trochlear nerve innervation, the lamprey caudal oblique is likely to be homologous to the gnathostome superior oblique. However, because the number of oculomotor and abducens nerve-innervated muscles differ between the two taxa, it is difficult to identify one-to-one correspondence of the muscles. Nishi [31] suggested that the lamprey ventral rectus is homologous to the gnathostome lateral rectus, and the lamprey caudal rectus corresponds to the additional EOMs, such as the retractor bulbi in gnathostomes. Nishi also suggested that there are two types of muscle duplication patterns of oculomotor nerve-innervated muscles in gnathostomes based on the branching pattern of the nerve. One is that the lamprey dorsal rectus corresponds to the superior rectus and medial rectus in gnathostomes: these muscles are innervated by the dorsal branch of the oculomotor nerve in sharks and lungfishes. The other is that the lamprey anterior oblique corresponds to the medial rectus and inferior rectus, and the lamprey anterior rectus corresponds to the inferior oblique (all innervated by the ventral branch of the oculomotor nerve) in the other gnathostomes. Based on a neurolabeling analysis, Fritzsch et al. [30] modified these ideas to indicate that the lamprey anterior oblique corresponds to the gnathostome inferior oblique, and that the gnathostome medial rectus evolved by duplication of the superior rectus in elasmobranchs and of the inferior rectus in osteichthyans. In the present study, we could not validate the one-to-one correspondence of EOMs, thus we retained the current nomenclature (see also Additional file 1). However, in the lamprey, the dorsal rectus (innervated by the dorsal branch) is differentiated from a cell population distinct from that of the anterior rectus and the anterior oblique (innervated by the ventral branch) (Figs. 5 and 6), providing a clue to determining the correspondence. Also in the chicken [22], there seems to be a close relationship between the medial rectus and inferior rectus, supporting the hypothesis that these two rectus muscles were duplicated in the osteichthyans including amniotes. Further detailed morphological studies on the EOM differentiation in various gnathostomes species are needed to confirm the one-to-one correspondence of EOMs.

Our findings indicate that the latest common ancestors of the vertebrates would have possessed three dorsal head mesodermal portions, although clear morphological segmentation may not have been present. Although the correspondence between the cranial nerves and the mesodermal components is suggestive of a somitomeric type of segmental organization, developmental regionalization and specification do not necessarily indicate the presence of mesodermal segments by themselves. The present data thus support neither presence nor absence of somite-like mesomeres in the ancestral head. Nevertheless, the ancestral dorsal head mesoderm would have also been specified by *Pitx2*, *Gsc* and *Tbx1/10*, and differentiated into EOMs, in a distribution pattern more or less similar to that seen in the modern lampreys.

It remains enigmatic how the head mesoderm and extrinsic eye muscles arose in the vertebrate ancestor. The data from the present study do not resolve this issue. Nevertheless, the present observations suggest that the dorsal head mesoderm is likely to have gone through tripartite regionalization during development by the time of the latest common ancestor of vertebrates. Further comparative developmental studies will be needed to gain insight into the origin of this vertebrate-specific embryonic structure.

## Conclusions

We conclude that the EOMs in lamprey developed from three components in the dorsal head mesoderm, which are genetically and regionally specified without segmental boundaries. This developmental mechanism is conserved among vertebrates, indicating that the tripartite origin of EOMs was established in the common ancestor of the vertebrates. Furthermore, our results support the hypothesis that the common ancestor of the vertebrates possessed lamprey-type EOMs, and that the disposition was modified secondarily in the common ancestral lineage of the chondrichthyans and osteichthyans.

### Nomenclature

- ao anterior oblique
- ar anterior rectus
- cb constrictor buccalis
- co caudal oblique
- cr caudal rectus
- dr dorsal rectus
- elv elevator labialis ventralis
- gV_1_ ophthalmicus profundus nerve ganglion
- gV_2,3_ maxillomandibular nerve ganglion
- gVII facial nerve ganglion
- hc hyoid cavity
- hm hyoid mesoderm
- io inferior oblique
- ir inferior rectus
- lr ateral rectus
- mc mandibular cavity
- mm mandibular mesoderm
- mr medial rectus
- oe oral epithelium
- pc premandibular cavity
- pm premandibular mesoderm
- pp phalangeal pouch
- rpe retinal pigment epithelium
- vr ventral rectus
- II optic nerve
- III oculomotor nerve
- IV trochlear nerve
- VI abducens nerve

## Additional file

Additional file 1. Homology and developmental patterning of EOMs. (XLS 29 kb)
